# Calcium Channels, OST1 and Stomatal Defence: Current Status and Beyond

**DOI:** 10.3390/cells12010127

**Published:** 2022-12-28

**Authors:** Santosh Kumar Upadhyay

**Affiliations:** Department of Botany, Panjab University, Chandigarh 160014, India; skupadhyay@pu.ac.in

**Keywords:** ABA, Ca^2+^, calcium channels, CNGC, OSCA, OST1, PAMP, stomatal defence

## Abstract

Stomatal immunity is regulated by pathogen-associated molecular patterns (PAMPs)- and abscisic acid (ABA)-triggered signalling in different ways. Cytoplasmic Ca^2+^ signature in the guard cells plays a vital function in stomatal immunity, but the mechanism of Ca^2+^ import is unknown. It has been very recently established that the hyperosmolality-gated calcium-permeable channels (OSCAs) and cyclic nucleotide-gated channels (CNGCs) are responsible for the influx of Ca^2+^ in the cytoplasm, which are activated after BIK1-mediated phosphorylation and ABA interaction during PAMPs- and ABA-triggered stomatal immunity in plants, respectively. Further, ABA-triggered OPEN STOMATA1 (OST1) causes the disassembly of microtubules in the guard cells besides activation of S-type anion channels (SLAC1) for the efflux of cytoplasmic anions that leads to stomata closure.

## 1. Introduction

Stomatal immunity is a natural process of plant defence during pathogens’ attack and abiotic stress [1]. Stomata closure is triggered by complex mechanisms involving pathogen-associated molecular patterns (PAMPs)- and abscisic acid (ABA)-associated signalling processes [2,3]. Both of these cascades lead to the elevation of cytoplasmic Ca^2+^ and reactive oxygen species (ROS), and activation of the S-type anion channel (SLAC1) in guard cells. SLAC1 efflux the cytoplasmic anions, thereby causing membrane depolarization and water loss, which ultimately leads to stomata closure [4]. Elevation in cytoplasmic Ca^2+^ concentration plays a central role in both PAMPs- and ABA-triggered stomatal defence that has been discussed earlier in several reviews [1,3]. However, the role of specific Ca^2+^ permeable channels in this process has been recently established in separate studies [5,6]. Further, the mechanism of disassembly of cortical microtubules during stamata closure has also been very recently described [7]. None of the available reviews have compiled these recent reports together for the better understanding of the mechanism of stomata closure. In the current communication, all these recent developments have been compiled and an inclusive mechanism of stomata closure along with the future perspectives have been highlighted. 

## 2. Mechanism of PAMPs-Triggered Stomata Closure 

The PAMPs-triggered stomatal immunity is mainly based on the signalling cascade started with the recognition of bacterial flagellin protein (flg22) by FLAGELLIN SENSITIVE2 (FLS2) receptor kinase in Arabidopsis [1,3]. The flg22 first binds with the extracellular leucine-rich repeat (LRR) domain of FLS2 and further interacts with the LRR domain of a coreceptor BRI1-associated kinase 1 (BAK1) that phosphorylates BOTRYTIS-INDUCED KINASE1 (BIK1). The BIK1 starts a phosphorylation cascade that ultimately activates the downstream pattern-triggered immunity (PTI) including MAP-kinase cascade, ROS burst, and influx of Ca^2+^ in the cytoplasm [8]. In addition, BIK1 is also involved in the phosphorylation of OPEN STOMATA1 (OST1), which activates SLAC1 for the exit of anions during the stomatal defence. Moreover, the autophosphorylation of OST1 is also well-known [9]. 

A study by Li et al. [10] established that BIK1 is essential for stomata closure by activating NADPH oxidase (RBOH) through phosphorylation at various Ser residues (Ser39, Ser343, and Ser347). However, the occurrence of EF-hand domain for Ca^2+^ binding, and Ca^2+^-dependent protein kinases (CDPK; CPK5) mediated phosphorylation indicate coordinated regulation of RBOH activation. In addition, OST1-mediated phosphorylation of RBOH is also reported [9]. The activation of RBOH is responsible for H_2_O_2_ production by ROS burst that ultimately leads to stomatal closure. However, partial response to exogenously supplied H_2_O_2_ in the *bik1* mutant of Arabidopsis suggested the necessity of other components in stomatal immunity [10]. Moreover, it is not known how H_2_O_2_ activates SLAC1 for the exit of anions from the guard cells during stomata closure. It might be associated with an unknown signalling mechanism to activate SLAC1 and/or solely involved in defence signalling to the adjacent cells, which should be validated in further research. 

**Figure 1:**
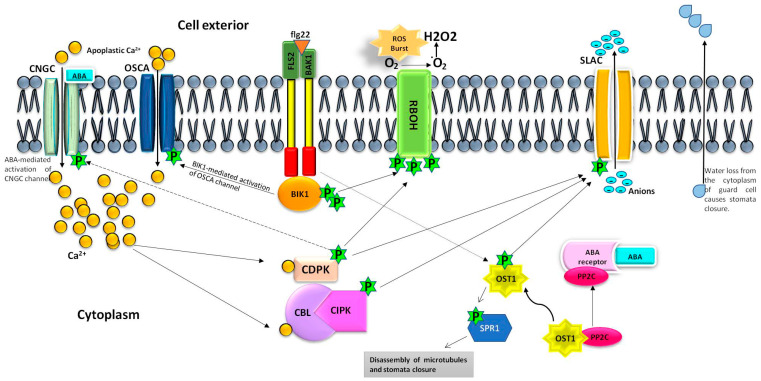
Calcium channels-mediated regulation of stomatal defence. Tan et al. [5] and Thor et al. [6] reveal the identification of Ca^2+^-permeable channels, belonging to cyclic nucleotide-gated channels (CNGCs) and hyperosmolality-gated calcium-permeable channels (OSCAs) associated with stomatal immunity. Abscisic acid (ABA) activates the CNGCs localized in the plasma membrane of guard cells for the influx of Ca^2+^ in the cytoplasm that leads to Ca^2+^-dependent stomatal closure by activating the S-type anion channel (SLAC1) [5]. However, the mechanism of ABA-CNGC interaction is still not known. In addition, ABA also releases OPEN STOMATA1 (OST1) from TYPE-2C protein-phosphatases (PP2C) after forming the ‘ABA-ABA receptor-PP2C complex’. The OST1 undergoes either autophosphorylation or transphosphorylation by BAK1 and directly activates SLAC1 that leads to the Ca^2+^-independent stomatal closure [3,9,15,17]. Further, Wang et al. [7] recently described OST1-mediated phosphorylation and dissociation of MAP SPIRAL1 (SPR1) from microtubules, thereby causing the disassembly of microtubules that leads to stomata closure during ABA-mediated stomatal immunity. Thor et al. [6] describe the role of OSCA channels (OSCA1.3 and OSCA1.7) in stomatal defence. The recognition of flagellin protein (flg22) by the leucine-rich repeat (LRR) domain of FLAGELLIN SENSITIVE2 (FLS2) and BRI1-associated kinase 1 (BAK1) receptors leads to the phosphorylation of BOTRYTIS-INDUCED KINASE1 (BIK1), which further phosphorylates the plasma membrane-localized OSCA channels in the guard cells, thereby activating them for the influx of Ca^2+^ from the apoplast to the cytoplasm [11]. This Ca^2+^ signature is possibly recognized by specific calcium-binding proteins/kinases that lead to the activation of SLAC1 by phosphorylation. SLAC1 facilitate the efflux of anions from the cytoplasm which ultimately leads to water loss and stomata closure. In addition, NADPH oxidase (RBOH) activation occurs by coordinated regulation through Ca^2+^ binding and phosphorylation by BIK1, CDPK, and OST1 that perhaps lead to H_2_O_2_-mediated signalling for stomatal defence, but the exact mechanism is not known.

Though the elevation of cytoplasmic Ca^2+^ concentration in the guard cells during stomatal defence is reported in various earlier studies [1,3,8], its mechanism of transport by specific Ca^2+^ channels was not known. Since the PAMPs-triggered stomatal defence starts with the phosphorylation cascade initiated by BIK1, Thor et al. [6] identified a Ca^2+^-permeable channel (OSCA1.3) specifically expressing in the plasma membrane of the guard cells and activated after phosphorylation. The BIK1 phosphorylates OSCA1.3 at the Ser residue (Ser 54) in a similar way to RBOH, and activates Ca^2+^ influx from the apoplast to the cytoplasm (Figure 1). They have also identified a homologous Ca^2+^ channel OSCA1.7 that also functions similarly. It is further established that these channels are specifically associated with stomatal immunity unlike the BIK1, which is also associated with general immunity through MAP kinase cascades. The specific expression of OSCA1.3 and OSCA1.7 in the guard cells, and their precise induction after PAMPs recognition, might be associated with stimulus-specific ‘calcium signature’ and deciphered with the help of specific calcium-binding proteins/kinases [11]. Since the OSCAs belong to the category of mechanosensitive ion channels due to their mechano-sensing nature [12,13], the hyper-osmotic response of these channels also needs to be analysed in future research, which is earlier reported as a basic function of OSCA channels [14].

## 3. Mechanism of ABA-Triggered Stomata Closure 

The ABA-mediated stomatal defence begins with the binding of ABA to its receptors (PYRABACTIN RESISTANCE (PYR/ PYL)/ABA RECEPTORS) that triggers the interaction of ABA receptors with TYPE-2C protein-phosphatases (PP2Cs). Since PP2C is responsible for the inhibition of OST1, the above interaction releases OST1 that activates SLAC1 by phosphorylation at Ser120 for the exit of anions from the cytoplasm of the guard cells and ultimately leads to stomata closure [3,15]. It was earlier thought that the ABA-signalling pathway is independent of Ca^2+^-signalling, because OST1 could directly activate SLAC1. However, an ABA-induced increase in cytoplasmic Ca^2+^ in the guard cells, activation of SLAC1 by CDPK through an alternate pathway, and only partial closing of stomata through ABA in the absence of Ca^2+^ suggested an interconnection between both the signalling pathway during stomatal immunity [15,16]. This interaction indicated the possibility of an ABA-activated Ca^2+^ channel in the guard cells. Moreover, OSCA Ca^2+^ channels, identified by Thor et al. [6], did not show any kind of interaction with ABA during PAMPs-triggered stomatal defence. 

To establish the connection, Tan et al. [5] examined the role of cyclic nucleotide-gated channels (CNGCs) by expression profiling in the guard cells, reporter assay and mutants analyses in the presence of ABA. This study reveals the enrichment of four CNGCs (CNGC2, 5, 6, 9, and 12) in the guard cells and their involvement in ABA-induced oscillation of cytoplasmic Ca^2+^ in Arabidopsis. However, only partial inactivity in stomatal movement in the *cngc* mutants suggested the positive involvement of other Ca^2 +^ channels, which might be OSCAs, in Ca^2 +^-mediated stomatal immunity. Further, CDPKs-mediated phosphorylation of CNGC6 also indicates the occurrence of any alternate pathway [17]. However, the interconnection among various Ca^2+^ channels and their mechanism of simultaneous activation at the time of stomatal defence needs to be validated in future studies. Moreover, ROS-induced Ca^2+^ signals could not be affected in the *cngc* mutants, which suggests the functions of these channels downstream to the ROS pathway [5]. However, the mechanism of interaction of CNGCs with ABA is a fundamental question. It is unclear whether ABA interacts with *cis*-acting regulatory elements or directly with CNGCs for activation, or it directs OST1-mediated activation of CNGCs by phosphorylation. These questions need to be addressed in future research.

Further, cytoskeleton reorganization is essential during stomatal movement, and the disassembly of microtubules in the guard cells is necessary for stomata closure. A recent study by Wang et al. [7] describes the OST1-mediated disassembly of cortical microtubules during ABA-mediated stomatal defence. The OST1 performs phosphorylation of MAP SPIRAL1 (SPR1) that dissociates it from microtubules, which leads to the disassembly of microtubules and stomata closure (Figure 1). However, Dou et al. [18] reported WAVE-DAMPENED2-LIKE7 (WDL7) as a negative regulator of microtubule disassembly that is degraded by MICROTUBULE-RELATED E3 LIGASE57 (MREL57) mediated ubiquitination during stomatal closure in ABA treatment. Hence, the exact mechanism is still a mystery. There might be coordination among the SPR1, WDL7, and MREL57 during ABA-mediated stomatal defence, but it requires further research. 

## 4. Conclusions and Future Perspectives

Taken together, the stomatal closure is a natural plant defence during both abiotic and biotic stress conditions. Though the initial steps of PAMPs- and ABA-induced stomatal defence are different, it is presumed to converge at OST1 and cytoplasmic Ca^2+^ rather than the SLAC1, which is activated in both Ca^2+^-independent and Ca^2+^-dependent manners after phosphorylation by OST1 and CDPKs/ calcineurin B-like (CBL)-interacting protein kinases (CIPKs), respectively [15,19]. This indicates a coordination in both of the mechanism of stomatal defence. Further, OST1 phosphorylates SPR1 and causes microtubule disassembly in the guard cells [7]. Both OSCAs and CNGCs Ca^2+^-permeable channels play vital functions in Ca^2+^ influx during stomatal immunity but their precise contribution is unclear, in terms of whether they work independently or in coordination. Furthermore, the actual mechanism of OST1 activation is also uncertain, in terms of whether it is activated by autophosphorylation after ABA-mediated detachment from PP2Cs or undergoes BAK1-mediated phosphorylation. Therefore, further investigation is necessary to decipher this complex and channelized mechanism.

## Data Availability

Not applicable.
